# Blimp-1 and systemic lupus erythematosus

**DOI:** 10.1186/ar4638

**Published:** 2014-09-18

**Authors:** Betty Diamond, Sun Jung Kim, Sebastian Schaetzle, George Georgiou

**Affiliations:** 1 Center for Autoimmune and Musculoskeletal Diseases, Feinstein Institute for Medical Research, Manhasset, NY, USA; 2 The University of Texas at Austin, TX, USA

## 

Our laboratory has been studying the impact of low Blimp-1 expression on dendritic cell function in SLE, analyzing both a mouse model of SLE in which there is a deletion of Blimp-1 in CD11c dendritic cells (DC Blimp-1^KO ^mice) and healthy individuals with the SLE risk allele of Blimp-1. This allele leads to reduced Blimp-1 expression in dendritic cells. Low expression of Blimp-1 alters cytokine expression following TLR activation and antigen-processing machinery. This leads to a highly distinct TCR repertoire in T-follicular helper cells in DC Blimp-1^KO ^mice. Much of this phenotype is under estrogen regulation. These data therefore provide an integrated explanation for altered cytokines, autoantibodies and female predisposition in SLE.

